# A Three-Dimensional Radiological Evaluation of a Nasopalatine Cyst by Cone-Beam Computed Tomography: A Case Report

**DOI:** 10.7759/cureus.117403

**Published:** 2026-10-03

**Authors:** Karthik Rajaram Mohan, Ramachandra Reddy Gowda Venkatesha, Kumar Appusamy, Reethika Rathan

**Affiliations:** 1 Oral Medicine and Radiology, Vinayaka Mission's Sankarachariyar Dental College, Vinayaka Mission's Research Foundation (Deemed to Be University), Salem, IND; 2 Oral Medicine and Radiology, Vinayaka Mission’s Sankarachariyar Dental College, Vinayaka Mission’s Research Foundation (Deemed to Be University), Salem, IND

**Keywords:** anterior maxilla, cone-beam computed tomography (cbct), marsupilization, nasopalatine duct cyst, three-dimensional imaging

## Abstract

A nasopalatine duct cyst (NPDC) is a rare fissural non-odontogenic cyst in the anterior maxilla region near the incisive papilla. The NPDC sometimes appears as an incidental finding in cone-beam computed tomography (CBCT). CBCT evaluates it in three-dimensional radiographic imaging planes, namely coronal, axial, and sagittal sections. The NPDC usually remains asymptomatic. Occasionally, patients complain of a sour or salty taste in the oral cavity. The genesis, hypotheses of its origin, radiographic characteristics in CBCT, diagnostics, treatment methods, post-surgical challenges, and clinical importance of NPDC are discussed.

## Introduction

A nasopalatine duct cyst (NPDC) is an oral fissural cyst of non-odontogenic origin that develops from remnants of the epithelium of the nasopalatine duct [1]. NPDCs comprise about 0.7% of all non-odontogenic cysts in the oral maxillofacial region. The NPDC was first described by Meyers [2]. The NPDC can occur asymptomatically and is discovered only during routine radiographic examinations [2]. Histologically, such cysts contain predominantly stratified squamous epithelium, pseudostratified and cuboidal epithelium with goblet cells, and cilia [3]. NPDCs are usually found near the incisive papilla region, where the nasopalatine canal opens [1]. NPDC occurs in 2.2% to 11.6%, or one in every 100 people [1]. The NPDC is caused by the persistence of epithelial remnants formed by the degeneration of the nasopalatine duct [3]. They occur in the fourth or sixth decades of life, with a slight male predominance [4]. Clinically, they are usually diagnosed by asymptomatic swelling posterior to the incisive papilla region on the midline region of the hard palate. Inflamed or infected cysts may cause symptoms such as pain, swelling, and discharge through the sinus opening in the patient [4]. The palatine papilla may form sinuses and drain due to an infection [4].

## Case presentation

A 20-year-old male reported for a routine radiological evaluation of his dentition. His past medical history revealed no systemic medical illness. His past dental history revealed no history of any previous trauma or surgery in the maxillary anterior teeth region. Intraoral clinical examination did not reveal the presence of dental caries, periodontal pockets, or mobility in the region of maxillary incisor teeth. The maxillary central incisors responded to the pulp vitality test, which revealed the absence of pulp vitality. A cone-beam computed tomography (CBCT) examination revealed the presence of an enlarged pear-shaped radiolucent lesion about the palatal aspect near the right maxillary central incisor region in continuation posteriorly with the nasopalatine canal and the presence of intact lamina dura without any evidence of any root resorption or periapical lesion (Figure 1).

**Figure 1:**
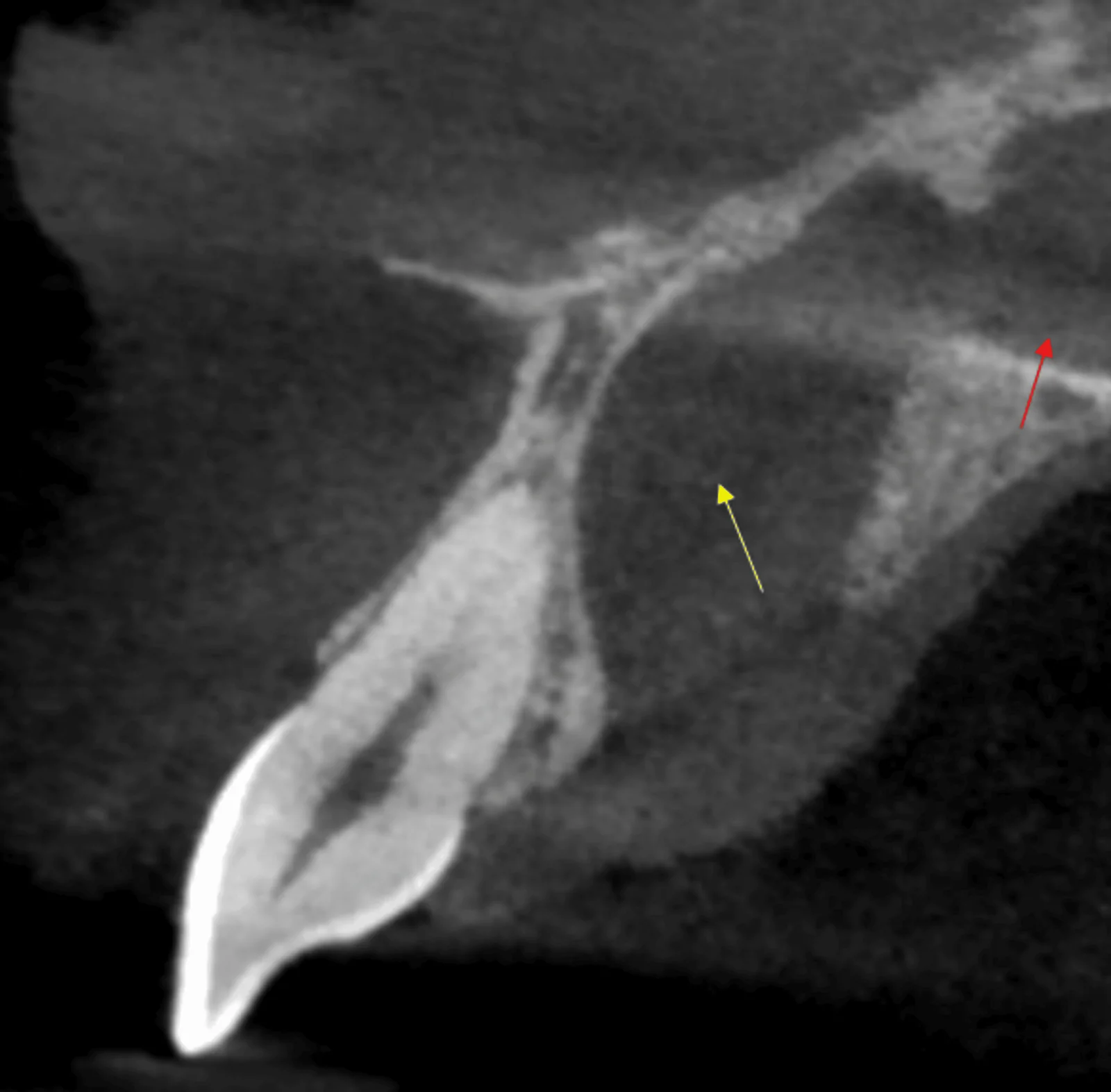
Cone-beam computed tomography (CBCT) sagittal section revealed a pear-shaped radiolucency (yellow arrow) in continuation posteriorly with nasopalatine canal (red arrow)

It was just noticed as an incidental finding in a routine radiographic examination. A well-defined pear-shaped radiolucency measuring about 12.8 mm x 10.4 mm involving the anterior maxilla region of the hard palate with an intact floor of the nasal cavity but a discontinuity in the hard palate is appreciated in the coronal section CBCT (Figure 2).

**Figure 2:**
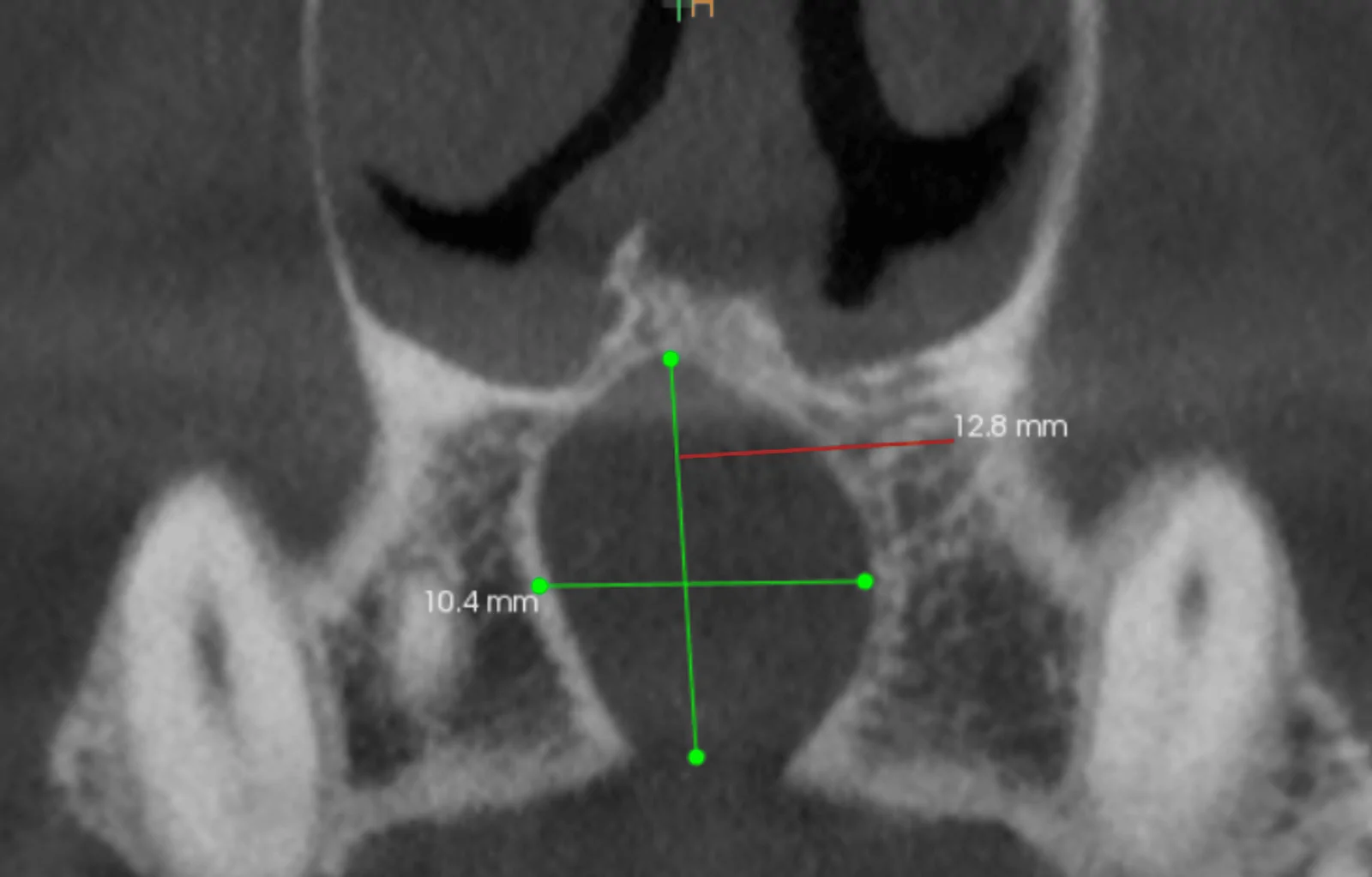
Cone-beam computed tomography (CBCT) coronal section revealed a well-defined radiolucency about 12.8 mm x 10.4 mm involving the anterior maxilla and intact floor of the nasal cavity but discontinuity of hard palate

The CBCT axial section revealed a well-defined round radiolucency of 7.6 mm x 7.3 mm in relation to the root of the right and left maxillary central incisors with intact lamina dura (Figure 3).

**Figure 3:**
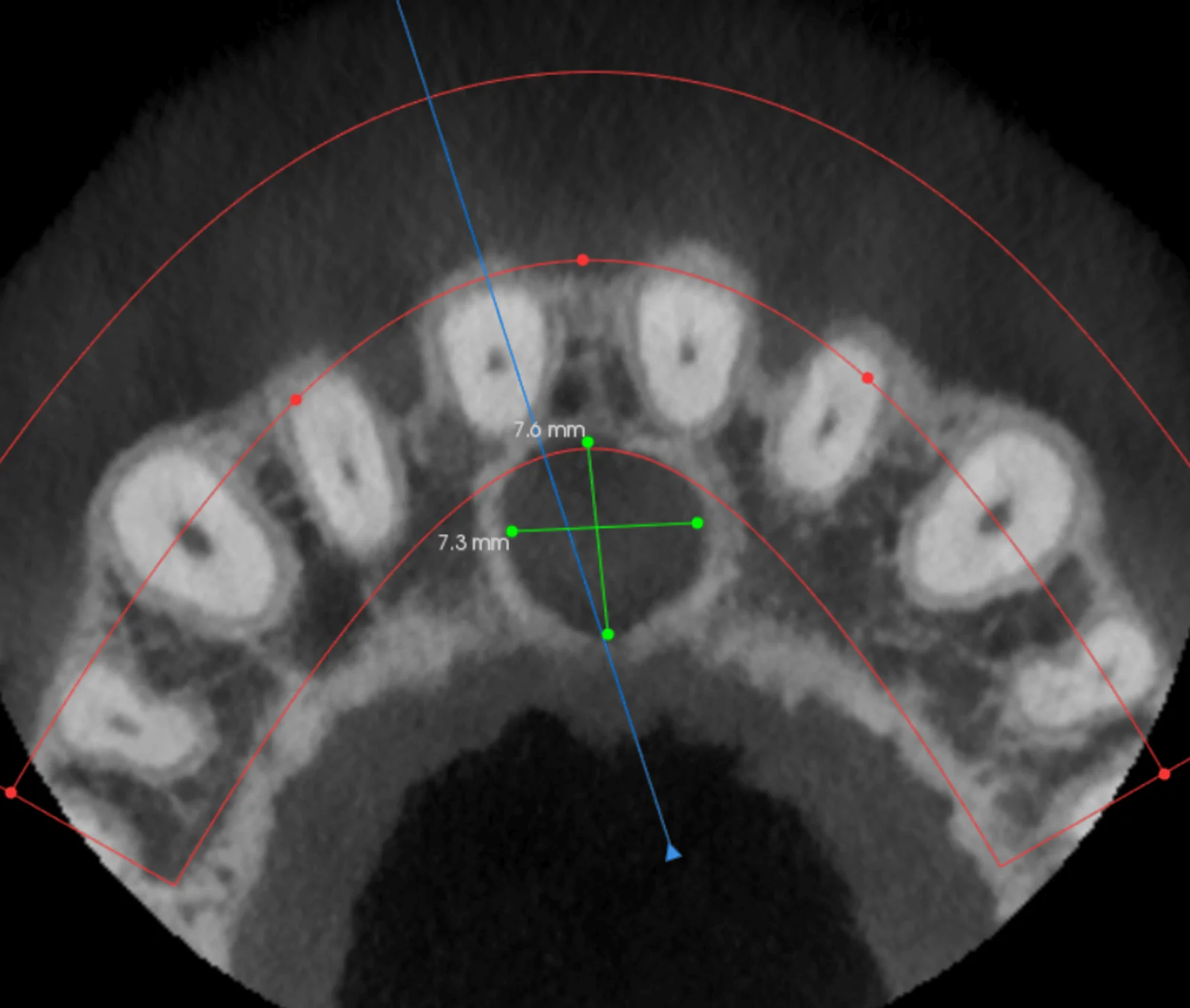
Cone-beam computed tomography (CBCT) axial section revealed a well-defined radiolucency measuring 7.3 mm x 7.6 mm in relation to the root of the maxillary central incisor

A three-dimensional reconstructed CBCT image revealed a defect in the lingual cortical plate in the hard palate in the vicinity of the maxillary central incisor (Figure 4).

**Figure 4:**
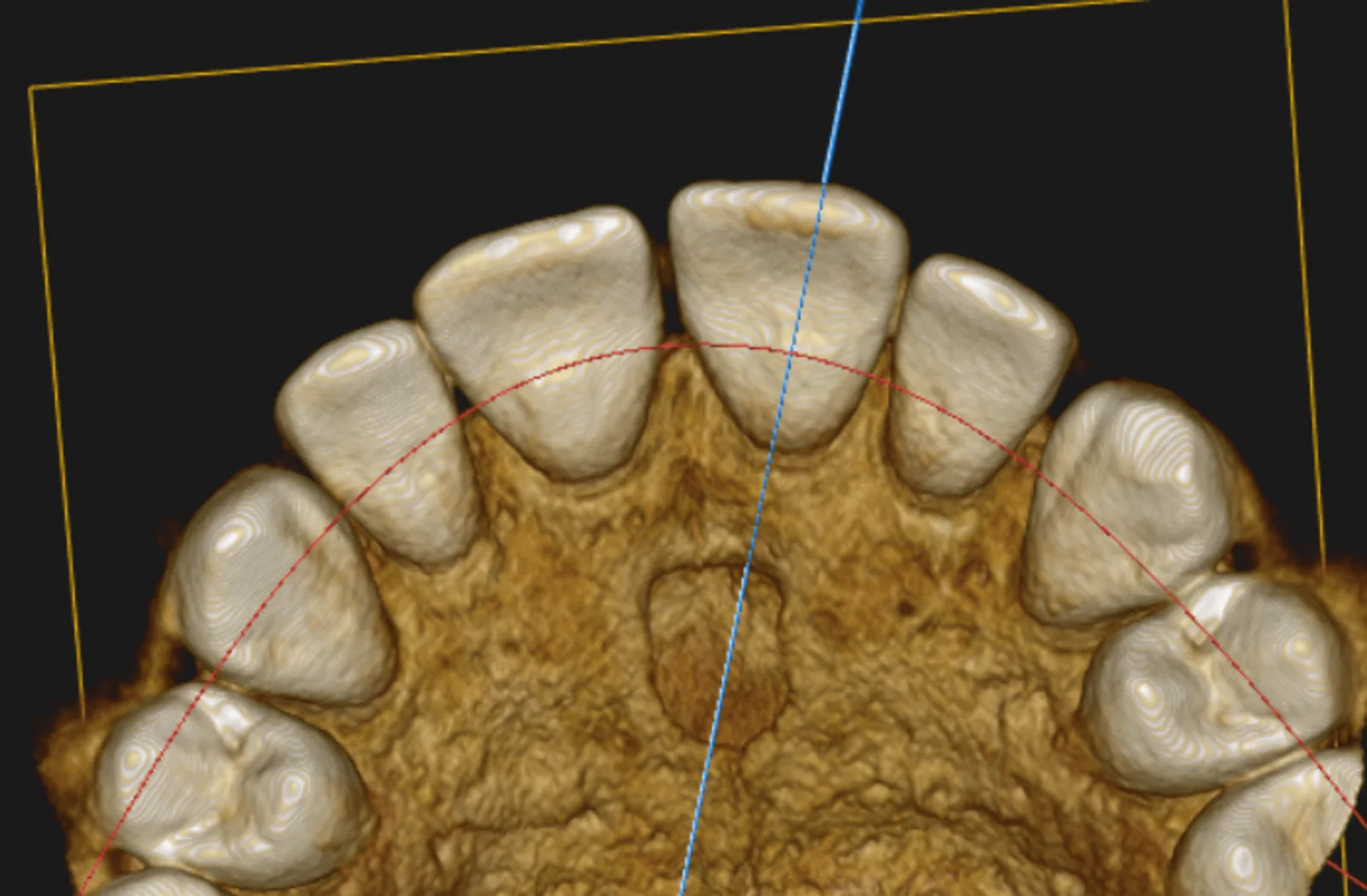
Three-dimensional reconstructed cone-beam computed tomography (CBCT) image revealed an oval-shaped defect on the hard palate in relation to the palatal aspect of the maxillary central incisors

Differential diagnosis

The differential diagnosis for NPDC includes periapical granuloma, which is differentiated by the presence of dental caries and no response to pulp-vitality tests, whereas the absence of dental caries in the NPDC. A radiolucent area in the midline of about 7.6 mm suggests the NPDC [1]. The incisive canal appears as a tube-like structure with a diameter of about 6 mm behind the maxillary central incisors in sagittal sections of CBCT. A radicular or periapical cyst appears as a well-defined radiolucency greater than 1.5 cm in diameter in the periapical root region of the maxillary central and lateral incisor regions [2]. The diameter of the well-defined radiolucent area measures less than 1.5 cm in diameter in periapical granulomas, and more than 1.5 cm indicates a periapical cyst [5]. Loss of lamina dura can occur in a periapical or radicular cyst. In radiographs, loss of lamina dura is appreciated in periapical granulomas and periapical cysts, whereas lamina dura is intact in NPDC. The tooth responds to pulp-vitality tests such as thermal or electric-pulp tests, whereas the radicular or periapical cyst does not respond to pulp-vitality tests. The nasolabial cyst clinically appears as a swelling in the nasolabial vestibule and clinically results in a stretched ala of the nose. NPDC appears as a localized intraoral swelling on the midline of the hard palate posterior to the incisive papilla region near the palatal aspect of the maxillary central incisors.

## Discussion

An NPDC is a rare fissural oral non-odontogenic cyst that develops from cystic degeneration and the proliferation of embryological remnants of the nasopalatine canal that contains nasopalatine nerves and vessels [1]. This cyst is called by other names such as "incisive canal cyst," "incisive duct cyst," "maxillary midline palatal cyst," "anterior midline cyst," and "anterior middle palatine cyst" [1]. The etiology of the NPDC is attributed to the entrapment of remnants of the epithelium of the oronasal duct within the incisive canal during the developing fetus, resulting in a NPDC. A direct blow to the incisive canal from trauma or ill-fitting dentures during mastication blocked ducts and accumulation of mucous glands [2]. The first theory states that the NPDC forms when epithelial cells remain within the mesenchyme after the medial and lateral nasal processes fuse. Based on the second theory, the cyst is thought to originate from epithelial remnants of the nasolacrimal duct [3].

Clinical features

NPDC is more commonly reported during the fourth to sixth decades of life. In our case, it was reported during the second decade of life. It is often asymptomatic and discovered as an incidental finding on a routine radiographic examination when a patient comes for dental screening. Some may complain of localized swelling in the hard palate posterior to the incisive papilla region. The swelling fluctuates; if superficially located, it has a bluish-tinge mucosa; deep-seated, the mucosa appears normal. The lesion can extend on one side of the nasal septum or near the maxillary labial frenum. Occasionally, the patient complains of sour or salty discharge [4]. The pressure caused by expanding NPDC results in paresthesia near the incisive papilla region or a burning sensation over the palatal mucosa.

Diagnostic modalities

The various diagnostic modalities for NPDC are described in Table 1.

**Table 1:** Diagnostic modalities of nasopalatine duct cyst (NPDC) Table created by the author.

Diagnostic modalities	Advantage	Disadvantage
Fibreoptic nasal endoscopy	To visualize and marsupialize the nasopalatine cyst	Requires pre-instillation of local anesthetic gel into nostrils
Contrast-enhanced computed tomography (CECT)	Three-dimensional visualization of nasopalatine duct cyst (NPDC)	Anaphylactic reactions to contrast medium
Magnetic resonance imaging (MRI)	Mickey Mouse sign in coronal sections of MRI [6]. Hyperintense T1 weighted signal due to the protein content.	Patients with ferromagnetic stents, drug aneurysmal clips, and cardiac pacemakers cannot undergo MRI scans claustrophobia. Noise generated during procedure
Cone-beam computed tomography (CBCT)	Highest spatial resolution. Helps to assess the three-dimensional extent of the NPDC. Lowest exposure (less than 36 microsieverts per exposure)	Low soft tissue detail

Radiographic features

The NPDC radiographically appears as an unilocular ovoid or concave-shaped radiolucency. Sometimes, it appears as a heart-shaped radiolucency due to the superimposition of the anterior nasal spine. CBCT must be considered in the radiographic diagnosis of the NPDC as it helps to view exact measurements in three-dimensional orthogonal planes, namely coronal, sagittal, and axial [4-6]. Some of the reported case studies on NPDC are listed in Table 2.

**Table 2:** The various case studies on nasopalatine duct cyst (NPDC)

Author	Year		No. of patients	Age (years)	Gender (M/F ratio)	Symptoms	Radiological findings	Size	Treatment
Anneroth et al. [7]		1986	32	23-88	M:F (1.9:1)	Swelling and pain	Round/ovoid	8-2.5 cm	Surgical enucleation
Norte and Wood [8]		1988	46	7-61	M:F (3:1)	Asymptomatic	Round	9-5.2 cm	Surgical enucleation
Allard et al. [9]		1981	22	11- 71	M=13	Intraoral swelling, pain	Round/ovoid/pear-shaped	5-22 mm	Marsupialization
Swanson et al. [10]		1991	334	9-84	M:F (1.2:1)	Intraoral swelling, drainage, pain	Ovoid	1.7 cm	Surgical enucleation
Takagi et al. [11]		1996	1	60	Male	Intraoral swelling with a sinus tract	Elliptical	30 mm	Partial resection of maxilla under general anesthesia
Ely et al. [12]		2001	1	8	Male	Swelling, pain	Heart-shaped radiolucency	1.5 x 2 cm	Surgical enucleation
Suter et al. [13]		2011	25	6-76	M:F (2.1:1)	Swelling, pain	Heart-shaped radiolucency	46.53 mm	Marsupialization
Barros et al. [14]		2018	30 out of 14564	17-90	M:F (1:1)	Swelling, pain	Ovoid radiolucency	2.37+/-1.69 cm	Surgical enucleation
Cavalcante et al. [15]		2021	45	43.2	M:F (1.1:1)	Asymptomatic	Ovoid radiolucency	2.1+/-1.1 cm	Surgical enucleation

Treatment

Transnasal endoscopic marsupialization is considered for the management of NPDC. Surgical enucleation is associated with a recurrence rate of 0% to 11% [15]. Post-surgical recurrence of NPDC is rare [16].

Complications

Complications include pain and paresthesia from nasopalatine nerve injury, infection, oro-antral fistula, pathological migration such as divergence and dehiscence of the root of maxillary incisors or associated impacted mesiodens, malignant transformation to squamous cell carcinoma, and sebaceous differentiation of NPDC [17-19]. The racial variation of the nasopalatine duct remains challenging for diagnosing such a rare NPDC [20].

Clinical significance

The clinical significance is that sound knowledge of the radiological findings alone guides the diagnosis of rare oral non-odontogenic cysts, such as NPDC. Oral non-odontogenic cysts, such as NPDC, are rare in the maxillofacial region and have varied morphology but typical radiological characteristics. Furthermore, a sound and thorough knowledge of the radiological findings of nasopalatine cysts in three-dimensional orthogonal planes such as coronal, axial, and sagittal sections of CBCT aids in diagnosing rare oral non-odontogenic cysts, such as NPDC, without the need of invasive procedures like histopathology and specialized methods such as immunohistochemistry staining.

In addition, this case report provides thorough knowledge about the three-dimensional radiological evaluation of the NPDC by CBCT, as one should be aware of the radiological findings of the nasopalatine duct cyst, which are usually overlooked and often missed in radiographic findings. Whenever a well-defined pear-, heart-, or oval-shaped radiolucency of more than 7.6 mm is encountered behind the palatal aspect of permanent maxillary central incisors, NPDC must be included in the differential diagnosis of other periapical pathologies such as periapical granuloma, cyst, or abscess.

Limitations

The main limitations of this case report were that intraoral clinical photographs of the patient were not provided, and post-treatment follow-up was not done as the patient feared the surgical procedure and did not provide consent to his intraoral clinical photographs and the surgical removal of the NPDC, as it was completely asymptomatic. Fine needle aspiration was not done in this case as the patient did not consent to the procedure since he was completely asymptomatic.

## Conclusions

NPDC is a rare non-odontogenic cyst often diagnosed by incidental findings during radiographic examination. The varied morphological and radiological appearances of NPDCs are often overlooked, and their diagnosis remains challenging for the clinician. CBCT offers better visualization of such NPDCs. CBCT is a mandatory tool for the diagnosis of NPDC. Dentists must be aware of the radiological findings of NPDC for precise diagnosis and to avoid unnecessary complications that can arise from invasive surgeries.
